# The burden of disease-specific multimorbidity among older adults in India and its states: evidence from LASI

**DOI:** 10.1186/s12877-023-03728-1

**Published:** 2023-01-30

**Authors:** Priyanka Patel, T. Muhammad, Harihar Sahoo

**Affiliations:** Department of Family and Generations, International Institute for Population Sciences, Mumbai, 400088 Maharashtra India

**Keywords:** Multimorbidity, Disease-specific burden, Older adults, India

## Abstract

**Background:**

Around the world, advances in public health and changes in clinical interventions have resulted in increased life expectancy. Multimorbidity is becoming more of an issue, particularly in countries where the population is rapidly ageing. We aimed to determine the prevalence of multimorbidity and disease-specific multimorbidity and examine its association with demographic and socioeconomic characteristics among older adults in India and its states.

**Methods:**

The individual data from the longitudinal ageing study in India (LASI) were used for this study, with 11 common chronic conditions among older adults aged 60 and above years (*N* = 31,464). Descriptive statistics were used to report the overall prevalence of multimorbidity and disease-specific burden of multimorbidity. Multinomial logistic regression has been used to explore the factors associated with multimorbidity.

**Results:**

Prevalence of single morbidity was 30.3%, and multimorbidity was 32.1% among older people in India. Multimorbidity was higher among females and in urban areas and increased with age and among those living alone. Hypertension, arthritis and thyroid were highly prevalent among females and chronic lung diseases and stroke were highly prevalent among males. The older people in the state of Kerala had a high prevalence of multimorbidity (59.2%). Multimorbidity was found to be more likely in older age groups of 75–79 years (RR-1.69; CI: 1.53–1.87) and 80 years and above (RR-1.40; CI: 1.27–1.56) and in the Western (RR-2.16; CI: 1.90–2.44) and Southern regions (RR-2.89; CI: 2.57–3.24). Those who were living with a spouse (RR-1.60; CI: 1.15–2.23) were more likely to have multimorbidity. Disease-specific multimorbidity was high in chronic heart disease (91%) and low in angina (64.8%).

**Conclusions:**

The findings suggest that multimorbidity has a positive relationship with advancing age, and disease-specific burden of multimorbidity is higher among chronic heart patients. Comorbidity, especially among those who already have chronic heart disease, stroke, cholesterol or thyroid disorder can have severe consequences on physical functioning, therefore, disease-specific health management needs to be enhanced.

## Background

Around the world, advances in public health and changes in clinical interventions have resulted in increased life expectancy. Significant demographic shifts have already occurred, and this trend will continue [1]. Still, the quality of life and functional capability have deteriorated due to non-communicable diseases that are strongly linked to ageing [2]. Worldwide, the population of people aged 60 and older is expected almost to double between 2015 and 2050, reaching around 2.1 billion [3]. The burden of chronic diseases has become a public health concern in low and middle income countries, with severe implications for primary and secondary care providers [4]. As a result of higher lifespan and higher exposure to risk factors for chronic diseases, the burden of multimorbidity is quickly rising in India. [5, 6] Multimorbidity, identified as two or more chronic conditions occurring in the same individual at the same time, is becoming more of an issue, particularly in countries where the population is rapidly ageing [7, 8], and it has been linked to a decrease in physical and mental functioning [7, 9] and reduced quality of life [10].

Assuming that a particularly prevalent chronic health problem, such as cardiovascular disease (CVD) or diabetes, may share various related risk factors, it is not uncommon for a single person to have more than one chronic illness. Studies in in western countries report that many people live with two or more chronic diseases due to an increase in life expectancy [11]. Similarly, multimorbidity (62.6%) is shown to be very common than single (18.8%) morbidity among middle-aged and older adult in India (age 45 years and above) [12] Rather than being the exception, multimorbidity is becoming normal [13]. However, several factors such as socioeconomic and demographic characteristics, alongside health and behavioural aspects are considered critical determinants of multimorbidity [7, 14]. In addition, a study shows a direct relationship between unhealthy dietary patterns, chronic disease risk factors and multimorbidity among women [15].

Further, multimorbidity shows a positive relationship with mental comorbidities, increasing with age in women and lower socioeconomic groups in Asian countries [16]. A previous study conducted in the Indian state of West Bengal found a 44% prevalence of multimorbidity [17], another study across the states of India found 42% multimorbidity prevalent in Kerala and 36% in Punjab [18]. Multimorbidity is more strongly predicted by age, economic independence, and lifestyle characteristics in the rural population in a study conducted in Bargarh district of Odisha [19].

There is substantial evidence on the regional variations in the prevalence of multimorbidity in India, attributed to differential healthcare access, utilization, under-reporting and under-diagnosis [20–22]. However, the burden of disease-specific multimorbidity and its state-wise prevalence and associated socio-demographic factors is an unexplored arena in India. In the current study, we aimed to assess the prevalence of multimorbidity and disease-specific multimorbidity and examine its association with demographic and socioeconomic characteristics in the older population. Disease-specific burden of multimorbidity was also estimated among older adults in India and its states by selected socio-demographic characteristics.

## Data and methodology

This study used data from the Longitudinal Ageing Study in India (LASI), which is a comprehensive nationwide survey that examines the health, economic, and social factors and effects of population ageing in India. The LASI collected data on the functional health, social and economic wellbeing, healthcare, burden of disease of older adults This survey is nationally representative of middle aged and older population in India and its states and union territories (UTs). Major states are considered to have more than 10 million per Census 2011. The LASI comprised a sample of 72,250 individuals aged 45 and above and their spouses from 35 Indian states and UTs, including 31,464 older people aged 60 and above and 6,749 oldest-old people aged 75 and above (excluding Sikkim). It is harmonized internationally with the Health and Retirement Study (HRS) and its sister studies across the world allow cross-national comparisons [23]. The data is publicly available and can be accessed by registration at https://iipsindia.ac.in/sites/default/files/LASI_DataRequestForm_0.pdf.

### Variable description

#### Outcome variable

In this study, outcome variable was chronic morbidity which is recorded as: no morbidity, single morbidity and two and above morbidity. Multimorbidity is the presence of two or more chronic conditions in the same individuals. For the current analysis, the following eleven chronic health conditions were included: hypertension, diabetes, cancer, chronic lung disease, chronic heart disease, stroke, arthritis, depression, cholesterol, thyroid and angina. In LASI, chronic disease was defined by self-report, the self-reported conditions were assessed based on responses to the question, “Have you ever been diagnosed with the following diseases?”.

#### Independent variables

The control variables were taken into consideration after extensive literature review. Sex of the respondent was available as male and female. Age-group was categorised into 60–64, 65–69,70–74, 75–79 and 80 years and above. Education was categorized into illiterate, primary, secondary and higher. Place of residence was available as rural and urban residence. Marital status was categorized into currently married and others (unmarried, divorced/ separated/ widow). Religion was categorized into Hindu, Muslim and others. Caste was categorized into SC/ST (Scheduled Caste/Schedule Tribe), OBC (Other Backward Classes) and others (including upper caste). Wealth quantile was categorized into five categories: poorest, poorer, middle, richer and richest. The geographical region was categorized into: north, south, east, west, north-east and central region. Tobacco use /smoking was categorised into yes/no. Living arrangement was categorized as living alone, living with spouse, living with children and living with others.

### Statistical analysis

**Fig. 1:**
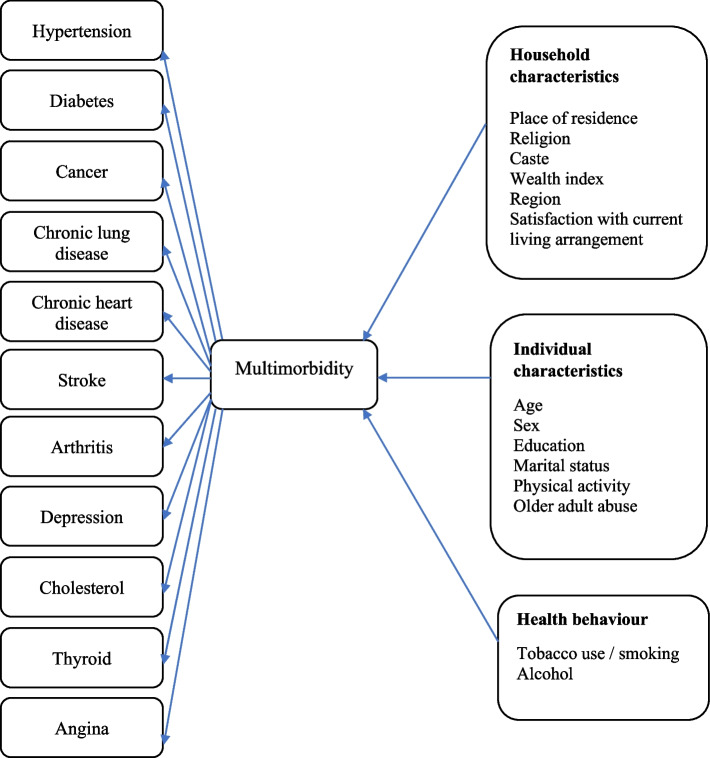
Conceptual framework for multimorbidity and its relationship with socio-economic variables

Descriptive analysis was conducted to report the prevalence and pattern of morbidity among older participants with different background characteristics. Chi-square test was used to examine the significance of the associations between sociodemographic variables and multimorbidity. A multivariate multinomial logistic regression models are performed to adjust for the possibly confounding impacts of other indicators (Fig. 1). In multivariate analysis, a multinomial logistic regression (MLR) [24] has been used to find out the factors associated with single and multimorbidity.

In MLR model, the estimate for the parameter can be identified compared to a baseline category. The logit models pair each response category with a baseline category, often the most common model is:

$$RR=\frac{P\left(Y=1\vert X+1\right)/P(Y=base\;category\vert X+1)}{P\left(Y=1\vert X\right)/P(Y=base\;category\vert X)}$$

The equation simultaneously describes the effects of X (independent variables) on the response categories, the effects vary according to the response paired with the baseline. The probability of each category can be calculated by the following equations:

$$\mathrm{P }\left(1:\mathrm{ single morbidity}\right)= \frac{\mathrm{exp}({b}_{0}+{b}_{1}{x}_{1}(2)+{b}_{n}{x}_{n}(2))}{1+\mathrm{exp}({b}_{0}+{b}_{1}{x}_{1}(2)+{b}_{n}{x}_{n}(2))}$$

$$\mathrm{P }(2:\mathrm{ multimorbidity})= \frac{\mathrm{exp}({b}_{0}+{b}_{1}{x}_{1}(3)+{b}_{n}{x}_{n}(3))}{1+\mathrm{exp}({b}_{0}+{b}_{1}{x}_{1}(3)+{b}_{n}{x}_{n}(3))}$$

$$\mathrm{P }\left(1:\mathrm{ no morbidity}\right)= 1-P\left(2:single morbidity\right)-P(3:multimorbidity)$$

where, P is the probability; ${b}_{0}$ denotes to the constant; ${b}_{1}$ and ${b}_{n}$ denote to the coefficients; and ${x}_{1}$ and ${x}_{n}$ denote to the number of independent variables.

The estimates of multinomial logistic regression in this study are presented in the form of Relative Risk (RR) and P is the probability of occurrences in the equation. Multinomial logistic regression was used with three categories of multimorbidity: i) no morbidity, ii) single morbidity and iii) multimorbidity. No morbidity was taken as base category and first category of independent variables was taken as the base category. The analysis was carried out in STATA-16 software. We further created a hierarchical heat cluster map and dendrogram using Origin software, to illustrate the disease-specific burden of multimorbidity across the states of India. Hierarchically-clustered heat map is a graphical representation of data, that uses colours to indicate values. The colour band of the cluster map gives information about the cluster map, such as the lower colour band indicating the lowest value of variable and the higher colour band indicating the greatest value of variable. The graph which is produced after doing agglomerative clustering on the data is called a dendrogram. The dendrograms on the sides demonstrate the independent clustering of the rows and the columns.

## Results

Table 1: The percentage distribution of sociodemographic characteristics among older adults in India was, over half (52.6%) of the older participants were females. A total of 70.6% of older adults belonged to rural areas. More than 50% of older adults were from the age group of 60–70-year. More than 50% of older adults were illiterate, 22% were primarily educated, 17% were secondary educated, and 3% were highly educated. Around 60% of older adults were currently married. The association of sociodemographic characteristics with morbidity among older adults. All of the sociodemographic characteristics were showing a significant relationship with multimorbidity. Multimorbidity was higher in females than males and higher in the urban area (42.7%) than in rural area (27.7%). The prevalence of multimorbidity and single morbidity increased with age, while slightly decreased in 80 and above age group. Single morbidity decreased with education while slightly increased in higher education. Multimorbidity increased with education while slightly decreased in higher education. Currently married women had less morbidity than others groups. Hindu and others religion had less multimorbidity than those from Muslim religion. SC/ST (24.7%) category had less multimorbidity than OBC (33.7%) and others category (39.7%). No much variation was observed in wealth groups for single morbidity but, multimorbidity increased in higher wealth quintiles. People from the north-east (35%) had high and those from the East (28.6%) had less single morbidity while, those from the south region (40.1%S) had high and those from central region (21.9%) had less multimorbidity. Those living alone had high (35.6%) and those living with others (29.4%) had less single morbidity while older adults living with children (34.5%) had high multimorbidity.

**Table 1:** Percent distribution and prevalence of multimorbidity among older adults in India by sociodemographic characteristics

Characteristic	N (%)	No Morbidity	Single Morbidity	2 & Above Morbidity
**Age group**				
60–64	9407 (29.9)	41.2	29.6	29.3
65–69	9003 (28.6)	36.9	30.1	33
70–74	5898 (18.8)	34.1	30.7	35.2
75–79	3603 (11.5)	32.6	31.1	36.3
80 +	3553 (11.3)	36.8	30.7	32.4
**Sex**				
Male	14931 (47.5)	40.6	29.9	29.7
Female	16533 (52.6)	35	30.7	34.5
**Place of residence**				
Rural	22196 (70.6)	42	30.3	27.7
Urban	9268 (29.5)	27	30.3	42.7
**Education**				
Illiterate	17783 (56.5)	41.5	31.1	27.4
Primary	7118 (22.6)	33.1	30.5	36.4
Secondary	5428 (17.3)	32.4	26.8	40.8
Higher	1135 (3.6)	30.1	32.8	37.1
**Marital Status**				
Currently married	19391 (61.6)	38.8	29.8	31.4
Others	12073 (38.4)	35.8	31.1	33.1
**Religion**				
Hindu	25871 (82.2)	37.7	30.6	31.6
Muslim	3548 (11.3)	31	28.4	40.6
Others	2045 (6.5)	40.1	29.6	30.3
**Social group**				
SC/ST	8505 (27)	45.3	30.1	24.7
OBC	14231 (45.2)	35.6	30.8	33.7
Others	8729 (27.7)	30.6	29.6	39.7
**Wealth Index**				
Poorest	6829 (21.7)	45	30.2	24.8
Poorer	6831 (21.7)	40.9	30.8	28.3
Middle	6590 (21)	38.7	31.2	30.1
Richer	6038 (19.2)	32.6	30.7	36.8
Richest	5175 (16.5)	28.2	28.2	43.7
**Region**				
North-East	935 (3)	39	35	26
Central	6593 (21)	48.5	29.7	21.9
East	7445 (23.7)	41	28.6	30.5
North	3960 (12.6)	35.6	32	32.4
West	5401 (17.2)	31.7	31.1	37.2
South	7130 (22.7)	29.3	30.6	40.1
**Tobacco/Smoke**				
Yes	12539 (40.2)	41.1	31	27.9
No	18665 (59.8)	35.3	29.9	34.8
**Alcohol**				
Yes	4555 (14.6)	42.4	30.7	27.0
No	26655 (85.4)	36.8	30.3	32.9
**Physical activity**				
None	10370 (33.2)	33.8	29.8	36.4
Moderate	11127 (35.7)	34.9	31.2	33.9
Vigorous	1645 (5.3)	40.9	31.2	27.9
Moderate Vigorous	8059 (25.8)	45.8	29.7	24.6
**Ill-treatment**				
Yes	1587 (5.2)	33.2	30.7	36.1
No	28840 (94.8)	38.2	30.2	31.6
**Satisfaction with current living arrangement**				
Strongly satisfied	6450 (21.1)	40.4	30.8	28.8
Satisfied	16428 (53.7)	37.8	30.7	31.5
Neither satisfied nor dissatisfied	5968 (19.5)	37.3	28.9	33.9
Dissatisfied	1454 (4.8)	31.2	29.8	39.0
Strongly dissatisfied	271 (0.9)	28.4	28.4	43.3
**Total**	**31464 (100)**	37.6	30.3	**32.1**

Table 2: The factors associated with multimorbidity are shown in Table 3. No morbidity was considered the reference category, and the first category was regarded as the reference category of independent variables. The female respondents were 1.3 times more likely to have single morbidity and 1.6 times more likely to have multimorbidity compared to male respondents. On the other hand, respondents from the urban areas were 1.2 times more likely to have single morbidity and 1.6 times more likely to have have multimorbidity than rural counterparts.

**Table 2:** Multinomial regression analysis of multimorbidity among sociodemographic variable among older adults in India

Characteristics	No morbidity (Base category)			
	**Single morbidity**		**Multimorbidity**	
	**RR**	**CI**	**RR**	**CI**
Age group				
60–64®				
65–69	1.08**	(1 1.2)	1.21***	(1.1 1.3)
70–74	1.33***	(1.2 1.4)	1.33***	(1.2 1.4)
75–79	1.32***	(1.2 1.5)	1.29***	(1.2 1.4)
80 +	1.21***	(1.1 1.3)	1.2***	(1.1 1.3)
Sex				
Male®				
Female	1.24***	(1.2 1.3)	1.46***	(1.3 1.6)
Place of residence				
Rural®				
Urban	1.34***	(1.2 1.4)	1.75***	(1.6 1.9)
Education				
Illiterate®				
Primary	1.21***	(1.1 1.3)	1.62***	(1.5 1.7)
Secondary	1.1*	(1 1.2)	1.71***	(1.6 1.9)
Higher	1.33***	(1.1 1.6)	1.48***	(1.2 1.8)
Marital Status				
Currently married				
Others	0.96	(0.9 1)	0.9***	(0.8 1)
Religion				
Hindu®				
Muslim	1.19***	(1.1 1.3)	1.41***	(1.3 1.6)
Others	1.03	(0.9 1.2)	1.21***	(1.1 1.4)
Social group				
SC/ST®				
OBC	1.04	(1 1.1)	1.11***	(1 1.2)
Others	1.14***	(1 1.2)	1.21***	(1.1 1.3)
Wealth Index				
Poorest				
Poorer	1.12***	(1 1.2)	1.27***	(1.2 1.4)
Middle	1.19***	(1.1 1.3)	1.37***	(1.3 1.5)
Richer	1.36***	(1.2 1.5)	1.88***	(1.7 2.1)
Richest	1.43***	(1.3 1.6)	2.55***	(2.3 2.8)
Region				
North-East®				
Central	0.71***	(0.6 0.8)	0.75***	(0.6 0.9)
East	0.81**	(0.7 1)	1.28***	(1.1 1.5)
North	0.99	(0.8 1.2)	1.34***	(1.1 1.6)
West	1.08	(0.9 1.3)	1.73***	(1.4 2.1)
South	1.1	(0.9 1.3)	1.83***	(1.5 2.2)
**Tobacco/Smoke**				
No®				
Yes	1.05	(1 1.1)	0.95	(0.9 1)
**Alcohol**				
No®				
Yes	1	(0.9 1.1)	0.9**	(0.8 1)
**Physical activity**				
None®				
Moderate	1.03	(1 1.1)	0.82***	(0.8 0.9)
Vigorous	0.86**	(0.8 1)	0.56***	(0.5 0.6)
Moderate Vigorous	0.83***	(0.8 0.9)	0.54***	(0.5 0.6)
**Ill-treatment**				
No®				
Yes	1.26***	(1.1 1.4)	1.52***	(1.3 1.7)
**Satisfaction with current living arrangement**				
Strongly satisfied®				
Satisfied	1.06*	(1 1.1)	1.19***	(1.1 1.3)
Neither satisfied nor dissatisfied	1.07	(1 1.2)	1.38***	(1.3 1.5)
Dissatisfied	1.37***	(1.2 1.6)	2.22***	(1.9 2.6)
Strongly dissatisfied	1.52**	(1.1 2.1)	2.94***	(2.2 4)

®-Reference category, *** if *p* < 0.01, ** if *p* < 0.05, * if *p* < 0.10, CI-Confidence Interval, RR-Relative Risk

**Table 3:** Prevalence of disease specific multimorbidity among background characteristics

Characteristic	Hypertension	Diabetes	Cancer	Chronic Lung disease	Chronic Heart disease	Stroke	Arthritis	Depression	Cholesterol	Thyroid	Angina
**Age group**											
60–64	72.7	79.2	76.5	73.9	94.1	83.6	68.4	76.6	91.2	90.5	61.0
65–69	74.0	86.1	78.0	68.5	90.7	89.5	71.7	76.7	96.1	87.1	66.8
70–74	72.6	82.5	88.1	81.7	94.5	83.9	71.1	87.3	97.9	77.1	63.4
75–79	69.1	84.1	95.8	77.5	79.1	77.8	68.3	82.3	85.6	86.5	66.3
80 +	64.7	88.3	70.8	75.8	89.7	79.4	73.4	89.5	53.1	94.6	70.7
**Sex**											
Male	73.1	80.1	81.1	69.8	88.0	84.4	63.6	80.9	81.9	82.9	63.5
Female	70.8	86.3	80.6	80.7	94.3	82.9	74.8	80.1	96.4	87.3	65.9
**Place of residence**											
Rural	69.9	81.7	72.6	72.9	89.3	78.5	68.3	79.7	90.8	83.4	60.7
Urban	74.3	84.7	90.2	80.8	90.9	94.3	74.8	86.3	88.3	88.3	78.1
**Education**											
Illiterate	68.4	83.7	71.4	72.6	89.2	78.2	68.0	80.2	95.1	78.3	59.8
Primary	73.8	81.8	86.2	75.1	90.7	90.3	69.2	86.2	93.1	94.2	70.8
Secondary	77.2	85.5	91.0	82.6	91.8	89.8	77.9	78.2	92.4	89.6	76.5
Higher	66.6	76.3	84.0	84.8	97.2	73.8	80.6	87.7	65.4	84.7	84.0
**Marital Status**											
Currently married	73.6	82.9	76.5	72.2	89.7	87.1	66.8	82.2	85.9	84.5	64.9
Others	67.2	84.2	86.1	79.5	93.5	78.6	75.2	80.7	95.9	88.9	64.7
**Religion**											
Hindu	71.6	82.4	80.0	74.8	90.7	83.5	69.6	81.3	86.7	88.3	63.5
Muslim	72.1	87.4	77.3	72.8	94.4	88.0	74.1	85.7	97.5	70.4	70.0
Others	72.4	86.9	92.9	85.1	86.5	79.8	73.6	76.9	93.4	95.9	69.9
**Social group**											
SC/ST	68.2	81.6	61.6	70.4	86.7	82.6	65.9	82.5	94.8	73.5	60.1
OBC	74.3	83.5	77.4	76.7	91.2	80.5	70.7	79.0	82.5	88.5	64.4
Others	70.3	83.6	89.9	77.3	92.6	88.4	73.7	84.6	95.2	88.6	70.5
**Wealth Index**											
Poorest	65.7	82.1	40.5	65.7	92.0	78.3	65.0	76.9	96.5	92.5	58.9
Poorer	66.3	81.9	75.9	70.3	91.2	82.3	63.7	91.5	97.3	86.9	63.1
Middle	69.3	80.3	64.5	74.5	82.4	75.6	72.1	78.1	66.9	86.4	63.6
Richer	76.3	82.5	93.6	78.8	89.4	93.4	71.1	82.4	92.4	79.1	67.2
Richest	78.7	87.6	78.3	84.9	96.6	88.6	79.8	79.1	90.7	91.0	72.5
**Tobacco/Smoke**											
Yes	71.8	79.8	66.8	70.9	87.4	82.9	65.3	79.9	89.0	92.4	60.3
No	71.5	84.6	86.9	78.5	92.9	84.2	73.3	82.7	89.0	83.8	68.3
**Alcohol**											
Yes	74.9	82.0	81.3	65.8	89.3	79.8	57.4	84.2	82.5	92.4	58.6
No	71.2	83.5	80.7	77.2	91.2	84.5	72.1	81.2	90.1	85.8	65.8
**Physical activity**											
None	74.2	84.0	79.6	75.9	92.6	82.1	70.6	84.1	96.6	79.1	72.8
Moderate	70.5	85.2	82.9	78.5	88.4	86.5	74.8	85.0	91.9	89.5	63.6
Vigorous	71.1	80.4	41.3	75.3	93.9	82.1	59.6	75.7	37.6	90.7	61.1
Moderate Vigorous	69.1	78.9	81.1	67.6	92.4	84.2	63.2	71.9	90.5	90.8	56.6
**Ill-treatment**											
Yes	80.3	92.5	91.0	80.2	68.9	80.0	70.5	81.6	86.6	93.4	66.7
No	70.9	82.9	79.4	75.0	92.4	83.9	71.0	82.4	88.9	84.9	64.2
**Satisfaction with current living arrangement**											
Strongly satisfied	67.5	77.0	83.6	75.5	92.8	86.8	68.5	81.2	75.1	82.1	63.7
Satisfied	69.8	83.4	82.8	74.1	89.8	81.6	67.8	83.2	96.2	86.6	65.1
Neither satisfied nor dissatisfied	78.0	87.1	76.1	76.4	91.9	81.0	76.5	78.5	90.0	87.8	61.2
Dissatisfied	78.8	90.4	51.9	78.3	93.3	94.9	74.1	80.3	89.7	97.5	72.7
Strongly dissatisfied	80.6	96.1	90.0	93.9	81.8	71.4	91.0	98.0	99.5	81.7	67.7
**Total**	**71.7**	**83.3**	**80.8**	**75.3**	**91.0**	**83.7**	**70.5**	**81.6**	**89.2**	**86.2**	**64.8**

The age group was significantly associated with multimorbidity. The people in the age group of 80 years and above were 1.4 times more likely to have multimorbidity compared to the 60–64-year age group. In case of education, older adults with primary, secondary and higher education were 1.5 times more likely to have multimorbidity compared to illiterate older adults. The wealth index was significantly associated with multimorbidity; 1.1 times in poorer, 1.2 times in the middle, 1.3 times in richer and 1.5 times in richest more likely to have single morbidity and 1.3 times in poorer, 1.5 times in the middle, two times in richer and 2.6 times in richest more likely to have multimorbidity than poorest category. In case of the region, it was more likely to have single morbidity, 1.2 times in the north, 1.3 times in west and 1.4 times in south. And it was more likely to have multimorbidity, 1.2 times in central, 1.7 times in the east, 1.9 times in the north, 2.2 times in west and 2.9 times in south as compared to the north-east region. The people who reported using tobacco/smoke were 1.1 times more likely to have single morbidity than not users. Those who were living with spouse were 1.5 times more likely to have single morbidity than living alone.

Table 3: shows the prevalence of disease-specific multimorbidity by sociodemographic characteristics. Some diseases had high prevalence of comorbidity. Females had high prevalence of disease specific multimorbidity like-diabetes (86.3%), chronic lung disease (80.7%), arthritis (74%) than male. Others social group had high prevalence of disease-specific multimorbidity of cancer (89.9%), chronic heart disease (92.6%) and stroke (73.7%) than SC/ST and OBC groups. Disease specific multimorbidity was high in those living with children in case of diabetes (87.5%) and chronic lung disease (80.2%).

Table 4: shows the prevalence of disease specific multimorbidity in India and its major states. Multimorbidity was higher in chronic heart disease (91%) followed by cholesterol (89.2%) and was lower in angina (64.8%) followed by arthritis (70.5%) at national level. In terms of specific disease of hypertension, multimorbidity was high in Kerala (83.7%) followed by Karnataka (80.8%) and lowest in Haryana (49.4%) followed by Jharkhand (56.3%). In terms of specific disease of diabetes, multimorbidity was high in Jammu & Kashmir (89.5%%) followed by Karnataka (88.4%) and lowest in Chhattisgarh (64.3%). Cancer multimorbidity was high in Tamil Nadu, Madhya Pradesh, Gujrat and Delhi (100%) and lowest in Chhattisgarh (0%). The multimorbidity of chronic lung disease was high in Karnataka (91%) and the lowest in Chhattisgarh (59.27%). The multimorbidity of chronic heart disease was high in Karnataka (98.23%) and the lowest in Chhattisgarh (78.19%). Strike multimorbidity was high in Punjab (97.8%) and lowest in Tamil Nadu (41.22%). Arthritis multimorbidity was high in Kerala (88.5%) and lowest in Chhattisgarh (53%). Depression multimorbidity was high in Kerala (94.32%) and lowest in Delhi (54.63%). Cholesterol multimorbidity was high in Uttarakhand, Uttar Pradesh, Telangana, Madhya Pradesh, Jharkhand, Jammu & Kashmir, Delhi, Chhattisgarh & Andhra Pradesh (100%) and lowest in Karnataka (57.46%). Thyroid multimorbidity was high in Chhattisgarh (100%) and lowest in Madhya Pradesh (57.76%). Angina multimorbidity was high in Kerala (92.84%) and lowest in Jharkhand (40.29%).

**Table 4:** Prevalence of Disease Specific Multimorbidity in India and its Major states

States	Hypertension	Diabetes	Cancer	Chronic Lung disease	Chronic Heart disease	Stroke	Arthritis	Depression	Cholesterol	Thyroid	Angina
India	**71.7**	**83.3**	**80.8**	**75.3**	**91.0**	**83.7**	**70.5**	**81.6**	**89.2**	**86.2**	**64.8**
Andhra Pradesh	76.6	87.9	80.0	76.3	87.7	91.8	72.7	75.0	100.0	88.9	76.9
Assam	59.1	86.3	81.1	83.5	89.2	94.7	82.6	89.2	82.4	63.7	53.5
Bihar	74.1	84.4	61.8	76.0	81.2	81.5	72.2	80.3	96.0	92.9	62.9
Chhattisgarh	65.2	64.3	0.0	59.3	78.2	74.9	53.0	62.2	100.0	100.0	45.6
Delhi	69.3	85.0	100.0	71.6	94.8	92.6	82.8	54.6	100.0	71.6	68.9
Gujarat	73.1	87.9	100.0	76.9	87.3	89.3	64.0	86.9	90.1	94.0	61.2
Haryana	49.4	78.0	71.1	61.4	84.6	58.6	70.2	68.1	74.0	81.8	77.2
Jammu & Kashmir	75.2	89.5	49.7	86.4	96.1	95.6	77.4	65.6	100.0	81.2	83.9
Jharkhand	56.3	72.8	67.1	63.6	85.1	85.0	56.7	66.5	100.0	93.5	40.3
Karnataka	80.8	88.4	87.4	91.0	98.2	81.8	73.3	85.0	57.5	97.9	61.8
Kerala	83.7	88.4	80.8	89.8	98.2	95.8	88.6	94.3	94.4	91.3	92.8
Madhya Pradesh	76.8	87.6	100.0	62.0	95.7	84.3	75.9	92.2	100.0	57.8	51.8
Maharashtra	74.8	84.9	76.1	85.0	89.2	82.6	66.9	80.1	97.3	88.3	74.6
Odisha	58.1	80.9	85.4	64.5	94.7	93.5	61.4	65.8	95.6	86.6	67.7
Punjab	70.8	81.4	88.3	82.9	88.9	97.8	77.5	85.5	96.7	73.5	85.1
Rajasthan	69.5	71.2	81.7	62.5	91.2	77.3	66.6	72.2	87.2	88.1	64.1
Tamil Nadu	75.7	77.3	100.0	71.2	93.7	41.2	63.5	73.3	73.7	82.4	74.1
Telangana	70.5	86.3	90.6	77.1	92.1	81.1	65.6	86.7	100.0	95.3	72.2
Uttar Pradesh	65.7	75.9	77.7	65.6	89.2	75.2	63.3	69.2	100.0	79.4	52.9
Uttarakhand	67.4	70.8	76.7	72.5	96.0	60.2	68.4	67.9	100.0	85.8	56.5
West Bengal	71.0	87.0	72.1	82.3	94.1	93.6	81.6	91.0	99.6	95.9	74.0

**Fig. 2:**
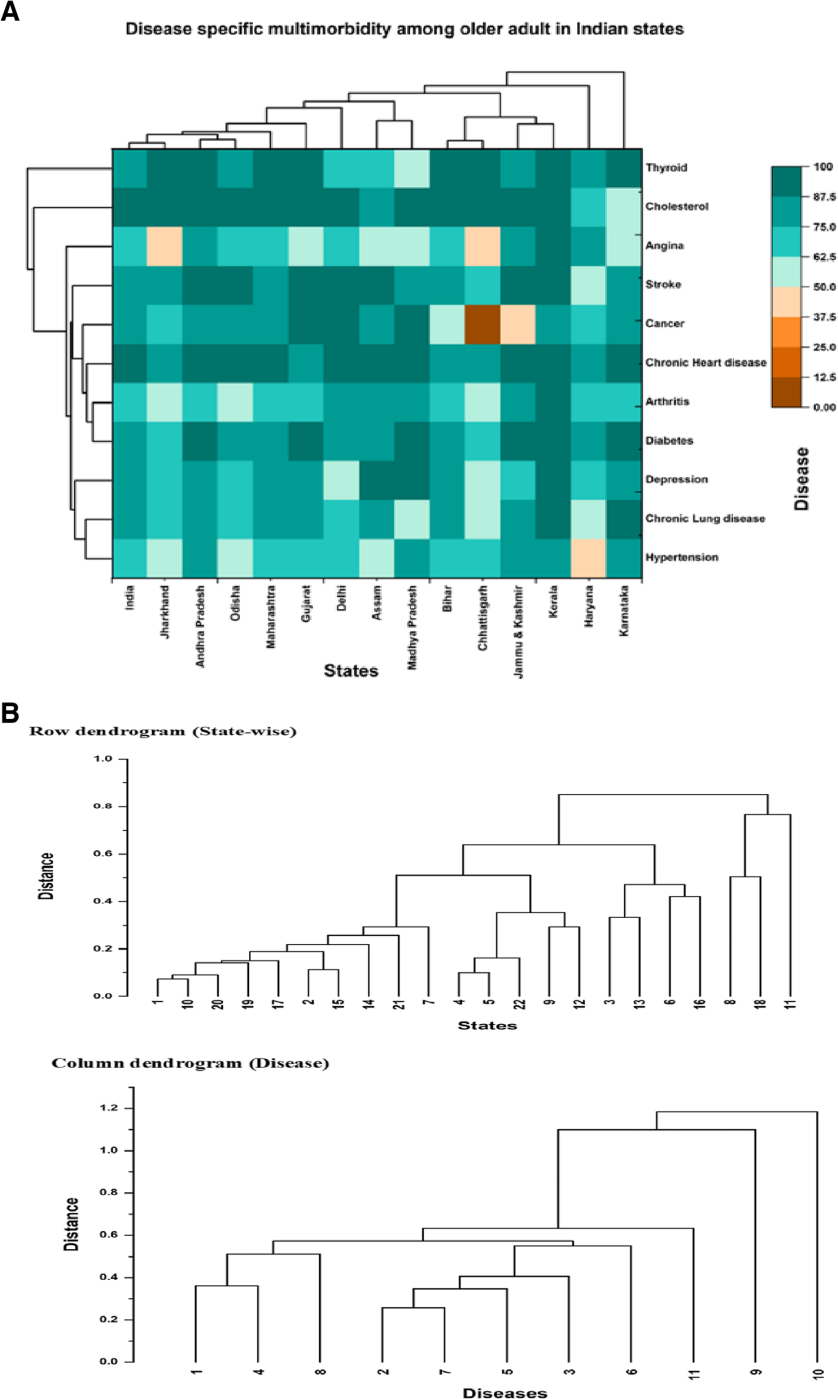
Disease -specific multimorbidty among older adult in Indian States. **a**. Heat map **b**. Dendrogram

The heat map shows the data value for each state and morbidity (Fig. 2a and b). Any patterns in the heat map may indicate an association between the state and morbidity. The colour band on the right side of the cluster map indicates information about the cluster map, such as dark orange showing the lowest disease specific multimorbidity and dark skyblue showing the highest disease specific multimorbidity among older adults in India. The height of dendrogram represent the distance between two cluster, higher the hight of dendrogram lower the similarity between the cluster and vise-versa. In our study, India and Bihar shows the same pattern of disease specific multimorbidity with euclidean distance (0.07) there after Odisha and Jharkhand show the second closet disease specific multimorbidity pattern with euclidean distance (0.09). Chronic lung disease and cancer, chronic lung disease and arthritis and hypertension and diabetes show the similar pattern of disease among different states in India with euclidean distance values (0.25, 0.34 and 0.35) respectively.

## Discussion

Globally, multimorbidity is arguably the most significant health care challenge with its wide range of adverse consequences. Despite multimorbidity being extensively studied, only a few research has looked at the combinations or patterns of morbidity in LMICs [25]. The present Study assessed the prevalence and correlates of multimorbidity and disease-specific multimorbidity among older people in India using the LASI dataset. Present study showed that approximately one-third of older population had single morbidity, and another one-third of them had multimorbidity in India.

Prevalence of multimorbidity in this study varies with that of other countries; it may be due to variation in socioeconomics, age pyramid, reporting of morbidity cases and health care system. For instance, a previous study n Germany showed that 62% older people over the age of 65 years were having multimorbidity [26], similarly, 45% of multimorbidity were found among people age > 65 years in Kosovo [27], 55% among those age above 77 years in Sweden [28] A study in Brazil found a prevalence of 29% of multimorbidity among older adults [29] while another study in Ghana found that 38.8% of outpatients had multimorbidity [30].

Further studies conducted in India in the past one decade showed the varying prevalence of multimorbidity across the states. A study in South India found a prevalence of morbidity in one-third of the population [31], another study in Odisha found less than one third prevalence of multimorbidity, where women had one-third and men had one fourth percent of multimorbidity [32]. Another study in India depicted that overall, 32% of respondents were having multimorbidity, among them 30.6% were multimorbid and among them 21.3% were people age 60–69 years [33]. In a study based on the LASI pilot survey, the average multimorbidity was 9%, whereas single illness was 17.4% among older Indian adults. Kerala had the highest rate of multimorbidity (30%), followed by Punjab (22.4%) [20]. The current Study also revealed a significant variance in single morbidity and multimorbidity among Indian states and regions.

The most often detected chronic conditions were hypertension and arthritis and it was shown to be the most often occurring comorbidities in studies in LMICs [19, 20, 34]. Some leading morbidities among Indian population reported in previous studies include diabetes, chronic lung diseases, arthritis and hypertension [35]. A primary study conducted in rural Tamilnadu showed that the, cataract (57%) was very common morbidity followed by bone and joint disease (43.3%). Other morbidities were, hypertension (14%), heart disease (9%), diabetes (8.1%) and asthma (6%) among older persons in rural Tamilnadu. Another study showed that overall, 63% of older adults suffered from at-least one non-communicable disease and 30.7% of them had multimorbidity [21]. Most common combination of morbidities were, high blood pressure and arthritis (7.5%), cataract and arthritis (5.3%) and high blood pressure and diabetes (4.7%). In our study, highest prevalence of comorbidity was hypertension followed by angina, arthritis, diabetes, chronic lung disease, chronic heart disease, depression, stroke, cholesterol, thyroid and lowest cancer.

Furthermore, it is documented that multimorbidity becomes more common progressively with age [21, 36–38]. In our study, morbidity was higher in the age group of 75–79 years and slightly decreased in the age group of 80 years and above. Again, multimorbidity was higher in females (34.9%) than males (29.7%), which is similar to previous findings and may be attributed to lower healthcare use, poor socioeconomic status, living and working environments and adverse life events [39, 40], Further, the socioeconomic disparities in the prevalence of multimorbidity were observed in this study. As nations get wealthier, the adoption of risky health behaviours tends to shift from higher to lower socioeconomic categories [41, 42]. According to several cross-sectional studies, multimorbidity is more prevalent among older individuals with lower educational and income levels [4, 43–45]. However, in our study, multimorbidity was highly prevalent among those belonging to the richest wealth quintile and those who are residing in urban areas. This suggests that the affluence of disease still exists in Indian context and the pattern has not changed in the country yet. On the other hand, the prevalence of multimorbidity was less prevalent among older adults who engaged in moderate or vigorous physical activity than those who were physically active, suggesting the protective effect of healthy behaviour on multiple disease prevalence. Nonetheless, alcohol and tobacco use were not associated with multimorbidity in this study. This may be due to the nature of variables which capture only ever use, suggesting the need for future research.

However, our findings confirm that the factors such as alcohol and tobacco consumption increase the risk of burden of majority of the disease-specific multimorbidity. This is particularly higher in case of non-communicable diseases such as cancer, chronic lung disease, chronic heart disease and angina. These findings have important policy implications. Notably, government policies have aimed to promote healthier lifestyles among individuals, but with limited success. Therefore, policy makers and health care providers should design effective health-promotion programs, especially among socioeconomically advantaged groups, who are found to be at increased risk of disease-specific burden of multimorbidity in this study. Thus, strategies to reduce the risk of multimorbidity may include facilitating more space for physical exercises, restricting marketing of tobacco and alcohol to avoid unhealthy behaviors and making smoking/drinking cessation services more accessible to the population; and conducting large-scale education campaigns among community-dwelling older persons to encourage healthier lifestyles.

Finally, our findings revealed large regional variations in disease-specific multimorbidity. Also, some diseases, in particular, have a high prevalence of multimorbidity in some states, which means that having multimorbidity indicates the increased presence of a particular disease occurring with other diseases. Around 91 percent of older people with chronic heart disease had multimorbidity. In terms of cholesterol, older persons from Uttar Pradesh, Delhi, and Bihar had higher burden of cholesterol-specific multimorbidity, whereas older adults in Rajasthan had higher prevalence of multimorbidity with chronic heart disease [46]. Moreover, higher burden of disease-specific multimorbidity was observed in case of chronic heart disease, stroke, cholesterol and thyroid disorder than other diseases. Among these, burden of chronic heart disease was highest in the states of Karnataka and Kerala. This may indicate the higher prevalence of unhealthy lifestyle among older people in the southern states of India, which calls for special attention in terms of policy and programs. Disease-specific policies should also focus on regional variations and target the subpopulations from states with higher burden of disease-specific multimorbidity.

### Limitations of the study

The present study has certain limitations. Due to the cross-sectional nature of the data used in our study, causation could not be established. Our study could only identify associations and longitudinal analyses may clarify the causal relationships among socioeconomic and demographic variables and multimorbidity. Also, our knowledge of the severity of morbidity and multimorbidity is constrained by the fact that the available statistics only provide information on prevalence and determinants. More importantly, the LASI did not include the institutionalized older adults who are expected to have more health problems and higher burden of multimorbidity than older adults in community-dwellings. Further, self-reported nature of several varriables including chronic conditions may lead to recall and reporting biases which might affect the current findings. The prevalence of diseases may be underestimated if some respondents are undiagnosed and may lead to biases, particularly if undiagnosed respondents are disproportionately of lower ocioeconomic status. Self-report of multimorbidity may also imply that some proportion of respondents who were diagnosed and treated with particular diseases may not have the disease at same time or at the time of survey.

## Conclusion

In conclusion, our study based on multimorbidity among older adults in India, shows 32% multimorbidity and 30% single morbidity. Multimorbidity was high in women and in urban areas. Since multimorbidity can have serious cognitive and functional consequences with growing older population in developing nations such as India, one of the important implications of our findings is that researchers and policy-makers should collaborate to develop effective intervention strategies, and health-promotional programmes and to train the health care personnel to minimize the disease-specific burden of multimorbidity. The findings suggest that multimorbidity has a positive relationship with advancing age, and disease-specific burden of multimorbidity is higher among chronic heart patients. Comorbidity, especially among those who already have chronic heart disease, stroke, cholesterol or thyroid disorder can have severe consequences on physical functioning, therefore, disease-specific health management needs to be enhanced. By enhancing public care facilities and increasing investments in the public health sector, policy steps should be adopted to encourage treatment seeking among older Indian population. Further, by introducing a specific policy push, the emphasis should be on the demands of geriatric healthcare, especially among the cardiovascular patients in Southern states of India. All parties involved, including the government, community health workers, and civil society, must play a crucial role in achieving this.

## Data Availability

The LASI datasets used in our study can be downloaded from the Gateway to Global Aging Data. Data are anonymized and available by request from the Gateway. To access the public release data from the Gateway to Global Aging Data, you must first register and get a username and password at- https://g2aging.org/login&r=%5Eq%5E.
